# The protective effect of chemical and natural compounds against amiodarone induced toxicity: A systematic review

**DOI:** 10.1016/j.bbrep.2026.102759

**Published:** 2026-09-06

**Authors:** Batool Zarei, Sepideh Elyasi, Gholamreza karimi

**Affiliations:** a Department of Clinical Pharmacy, School of Pharmacy, Mashhad University of Medical Sciences, Mashhad, Iran; b Pharmaceutical Research Center, Institute of Pharmaceutical Technology, Mashhad University of Medical Sciences, Mashhad, Iran; c Department of Pharmacodynamics and Toxicology, School of Pharmacy, Mashhad University of Medical Sciences, Mashhad, Iran

**Keywords:** Amiodarone, Toxicity, Pulmonary fibrosis, Oxidative stress, Mitochondria

## Abstract

**Objectives:**

Amiodarone remains an important antiarrhythmic drug, but cumulative exposure can cause pulmonary, hepatic, renal, thyroid, and reproductive toxicity. This systematic review critically compares interventions tested to prevent or attenuate amiodarone-induced injury, evaluates evidence strength and replication, and integrates the implicated molecular pathways.

**Methods:**

PubMed, MEDLINE, Scopus, and Web of Science were searched from inception to July 2025 for English-language in vitro, animal, and human studies of protective interventions against amiodarone toxicity. Animal studies were evaluated using SYRCLE; in vitro studies using an adapted OHAT framework; and the human observational study using the applicable JBI checklist. Because of substantial heterogeneity, findings were synthesized narratively by organ, mechanism, replication, and translational readiness. Database-specific search strategies are reported in Supplementary Table S1. Disagreements were resolved by consensus; agreement was not quantified.

**Key findings:**

Thirty-five reports were included. The evidence was overwhelmingly preclinical; only one observational human report was identified and no randomized clinical trial was found. Pulmonary models predominated. Vitamin E had the broadest replication, while curcumin, silymarin, l-carnitine, and grape-seed preparations were evaluated in more than one report or complementary model. Protection converged on attenuation of lipid peroxidation and inflammatory signaling, preservation of endogenous antioxidants and mitochondrial function, and suppression of apoptosis or TGF-β/Smad-associated fibrosis. Most studies had unclear risk of selection or performance bias.

**Conclusion:**

Current evidence establishes biological plausibility but not clinical effectiveness. No protective adjunct can presently be recommended for routine use with amiodarone. Independent replication, clinically relevant exposure schedules, pharmacokinetic-interaction testing, and rigorously designed human studies are required.

## Highlights

• Oxidative stress is central to amiodarone-induced toxicity.
• Natural compounds reduce amiodarone-induced organ injury.
• Antioxidants preserve mitochondrial and cellular function.
• Antifibrotic agents may attenuate pulmonary toxicity.
• Clinical evidence remains limited despite promising findings.

## 1 Introduction

Amiodarone is a potent class III antiarrhythmic drug used to treat supraventricular and ventricular tachyarrhythmias, including atrial fibrillation. Its high lipophilicity, variable absorption, large volume of distribution, and exceptionally long terminal half-life support durable antiarrhythmic effects but also promote tissue accumulation and delayed toxicity [1,2].

Adverse drug reactions related to amiodarone are numerous, affect multiple systems, and are often serious. Cardiac toxicities include suppression of sinus node function, atrioventricular block, and significant QT interval prolongation, all of which increase the risk of torsade de pointes, a life-threatening ventricular arrhythmia. Hypotension may occur during intravenous administration, often due to excipients rather than the active drug. Rare but severe hypersensitivity reactions, such as angioedema and anaphylaxis, have also been reported [[3], [4], [5]].

Pulmonary toxicity is one of the most feared complications, closely linked to cumulative dose and treatment duration. Clinical signs vary from interstitial pneumonitis to acute respiratory distress syndrome (ARDS), with the latter having mortality rates exceeding 50% in ventilated patients. Pathogenesis involves both cytotoxic and immune-mediated mechanisms, and risk increases in old individuals and those with pre-existing lung disease [[6], [7], [8]].

Hepatic involvement is common and can range from mild, asymptomatic transaminase elevation to chronic hepatitis, cirrhosis, and rarely, acute hepatic failure. Intravenous formulations have been associated with fulminant hepatotoxicity that mimics ischemic hepatitis, highlighting the importance of regular liver function monitoring during treatment [[9], [10], [11], [12]].

Ocular complications are also common. Corneal microdeposits appear in nearly all long-term users, usually without symptoms but sometimes causing visual halos or blurring. More serious issues include rare cases of optic neuropathy and optic neuritis, which can lead to permanent vision loss. Other signs include papilledema, macular degeneration, and periocular photosensitivity reactions [13].

Dermatologic toxicities greatly affect the quality of life. Photosensitivity is common, reported in nearly half of patients, while long-term use can lead to blue-gray skin discoloration. Serious but rare complications include toxic epidermal necrolysis, exfoliative dermatitis, and vasculitis, which can be life-threatening [14,15].

Endocrine dysfunction is significant due to the drug's iodine content. Amiodarone-induced hypothyroidism is more frequent in iodine-sufficient regions, while thyrotoxicosis dominates in iodine-deficient areas. Both conditions present major challenges for managing arrhythmias and can destabilize cardiovascular function in at-risk patients [16,17].

Neurological and neuromuscular toxicities of amiodarone include tremor, ataxia, neuropathy, and myopathy, occasionally causing severe functional impairment [18]. Psychiatric effects, though uncommon, may manifest as delirium, hallucinations, or depression [19]. Less frequent but clinically relevant complications involve reproductive, hematologic, and electrolyte disturbances, underscoring the drug's broad systemic toxicity profile [2].

Given amiodarone's indispensable role and its multi-organ toxicity, protective strategies are clinically relevant. Previous reviews have largely addressed incidence, clinical presentation, monitoring, mechanisms, and management. They have not systematically compared compounds tested specifically for protection, determined which findings have been independently replicated, distinguished preclinical from clinical evidence, or assessed translational readiness across organs. This review therefore evaluates the comparative strength of protective-intervention evidence, identifies recurrent mechanisms and independent replication, and defines the evidence required before clinical translation.

## 2 Materials and methods

### 2.1 Search strategy and information sources

This systematic review was conducted and reported in accordance with the Preferred Reporting Items for Systematic Reviews and Meta-Analyses (PRISMA 2020) guidelines. A comprehensive literature search was performed to identify original studies investigating the protective effects of chemical and natural compounds against amiodarone-induced toxicity.

PubMed, MEDLINE, Scopus, and Web of Science were searched from database inception to July 2025. Reference lists of eligible studies and relevant reviews were screened manually. Controlled vocabulary, where available, and free-text terms covered amiodarone, toxicity or organ injury, and protective or preventive interventions. Database-specific reproducible strategies, interfaces, coverage, and search dates are reported in Supplementary Table S1.

The search strategy combined Medical Subject Headings (MeSH) and free-text terms related to amiodarone, toxicity, organ injury, and protective interventions. The main search concepts included “amiodarone”, “toxicity”, “protective effect”, “preventive therapy”, “prophylaxis”, “kidney injury”, “lung injury”, “pulmonary toxicity”, “drug-induced liver injury”, “testicular injury”, and “thyroid disease”.

### 2.2 Eligibility criteria

Studies were considered eligible for inclusion if they met the following criteria: (i) original research articles employing in vitro, in vivo, or clinical study designs; (ii) investigations evaluating the protective effects of chemical, synthetic, biological, or natural compounds against amiodarone-induced toxicity; (iii) studies reporting at least one relevant outcome related to toxicity attenuation, including biochemical, histopathological, molecular, cellular, physiological, or clinical parameters; and (iv) articles published in English.

Studies were excluded if they met any of the following criteria: (i) review articles, systematic reviews, meta-analyses, editorials, letters to the editor, conference abstracts, case reports, or book chapters; (ii) duplicate publications; (iii) studies not specifically addressing amiodarone-induced toxicity; (iv) studies that did not evaluate a protective or preventive intervention; (v) publications lacking sufficient methodological information or outcome data for meaningful evaluation; or (vi) articles published in languages other than English.

### 2.3 Study selection

All records identified through the database search were imported into EndNote X9 software (Clarivate Analytics, Philadelphia, PA, USA), and duplicate records were removed. Two reviewers independently screened the titles and abstracts of all retrieved records according to the predefined eligibility criteria. Potentially relevant studies were subsequently subjected to full-text assessment.

**Fig. 1:**
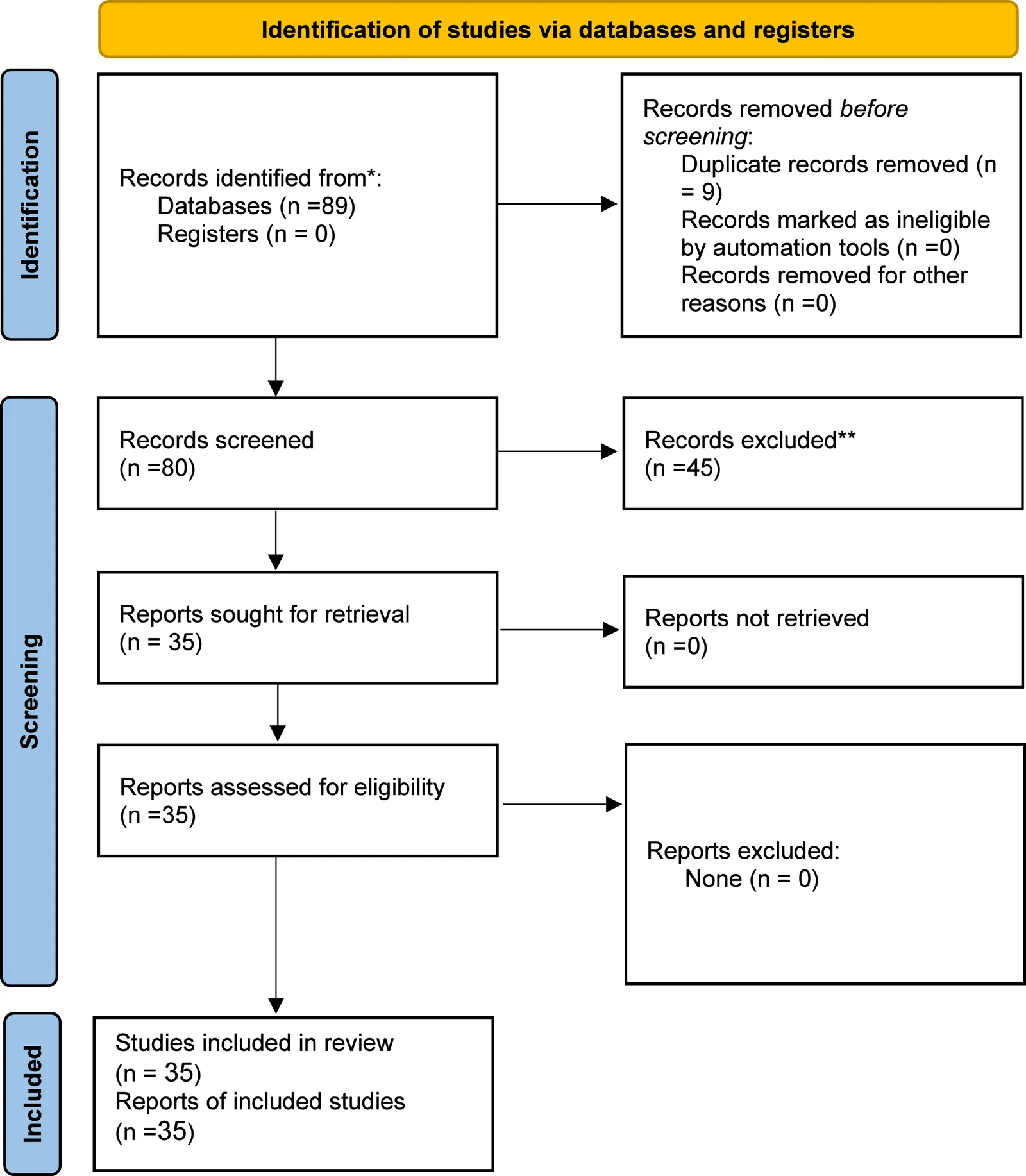
PRISMA flow diagram of the literature search and study selection for the systematic review.

Two reviewers independently assessed potentially eligible full texts. Disagreements were resolved through discussion and consensus. Agreement before consensus was not quantified; this is acknowledged as a methodological limitation. The selection process is shown in Fig. 1.

### 2.4 Data extraction and data synthesis

Data extraction was independently performed by two reviewers using a standardized data extraction form. The following information was collected from each eligible study: first author, year of publication, country of origin, study design, experimental model (cellular, animal, or clinical), target organ or tissue, amiodarone dose and exposure duration, type and dosage of the protective intervention, treatment duration, outcome measures, proposed mechanisms of action, and principal findings.

Discrepancies in data extraction were resolved through discussion and re-evaluation of the original articles. Given the substantial heterogeneity among the included studies with respect to experimental models, interventions, outcome measures, and target organs, a quantitative meta-analysis was not considered appropriate. Therefore, the findings were synthesized narratively according to the affected organ system and the proposed mechanisms underlying the protective effects of the investigated compounds.

Because outcome definitions, measurement scales, and reporting formats differed substantially across studies, no common standardized effect measure or pooled estimate was calculated. For the narrative synthesis and Table 1, findings were summarized according to the direction of change in the reported outcomes relative to the amiodarone-treated comparator, as reported by the original study authors. Effect magnitudes were not compared across studies (see Table 2).

**Table 1:** Summary of synthetic chemicals and natural compounds in mitigating AMD-induced toxicities.

Drug name	Amiodarone dose/route	Experimental model	Drug dose/route	Results	Reference
Adenosine Triphosphate	50 mg/kg/day oral for 14 days	In vivo Male albino Wistar-type rat	2 mg/kg and 5 mg/kg i.p for 14 days	↓oxidative stress, Inflammation in liver tissue ↑antioxidant	[20]
Chard (*Beta vulgaris* L. var. cicla)	100 mg/kg/day oral for 7 days	In vivo Male-Sprague-Dawley rats	500 mg/kg/day oral for 7 days	↓ oxidative stress, TNF-α, LFT ↑antioxidant,	[21]
Diacerein	100 mg/kg/day oral for 21 days	In vivo Wistar albino female rats	50, 100 mg/kg/day oral for 21 days	↓ LFT, MDA ↑Nrf2, GSH, inactivation of the TLR4/NF-\kappaB/NLRP3 pathway	[22]
Ginkgo Biloba	40 mg/kg/day oral for 8 weeks	In vivo white male albino rat	100 mg/kg/day oral for 8 weeks	↓ LFT, DNA damage, improvement in histopathological observations, ↑ Content of glycogen in the liver	[23]
*Glycyrrhiza glabra*	300 mg/kg/day oral for 2 weeks	In vivo male albino rats	200 mg/kg,400 mg/kg, 800 mg/kg for 2 weeks	↑antioxidant ↓lipid peroxidation, modulation inflammation	[24]
Grape seed	40 mg/kg/day oral for 8 weeks	In vivo white male albino rat	100 mg/kg/day oral for 8 weeks	↓lysosomal phospholipidosis, DNA damage, oxygen free radical, LFT	[23]
Livogrit	In vitro: 5 μg/mL by supplementing it directly into the cell culture medium In vivo: 100 μg/mL by supplementation in the NGM agar plates	In vivo: The nematode *Caenorhabditis elegans* In vitro: human liver (HepG2) cells	In vivo: 3, 10, and 30 μg/m supplementing it directly into the cell culture medium In vitro, 10, and 30 μg/m by supplementation in the NGM agar plates	↑ SOD, catalase, GSH, and GST, ↓ phospholipid accumulation	[25]
*Mangifera indica* L. stem bark	10 mM for 24 h	In vitro: human hepatoma HepG2 cell line	Pretreatment: 5, 50, or 100 μg/ml for 24 h before amiodarone Co-treatment: 5, 50, or 100 μg/ml for 24 h with amiodarone	↑antioxidant status, stabilization of mitochondrial membrane potential, regulation of intracellular Ca^2+^ levels	[26]
Melatonin	70 mg/kg/day oral for 4 weeks	In vivo male Wistar rats	50 mg/kg/day IP for 4 weeks	↓ LFT, LDH, γ-GT, p53 expression, and oxidative stress	[27]
Resveratrol	50 mg/kg/day oral for 2 weeks	In vivo male albino Wistar-type rats	25 mg/kg/day oral for 2 weeks	↓AST, caspase-3, and TNF-α	[20]
Silymarin	30 mg/kg/day oral for 8 weeks	In vivo albino rats	140 mg/kg/day oral for 8 weeks	↓MDA, TNF-α, IL-6, and LFT, ↑ SOD	[28]
Silymarin	150 mg/kg/day oral for 3 weeks	In vivo male Fischer 344 rats	60 mg/kg/day oral for 3 weeks	↓Amiodarone levels, LFT, MDA, TNF-α, and IL-6 ↑ SOD	[29]
Vitamin E	150 mg/kg/day oral for 3 weeks	In vivo male Fischer 344 rats	100 mg/kg/day oral for 3 weeks	↓Conjugated diene, TBARS, ↓number, and size of pathological lysosomes in liver tissue	[29]
Zizyphus Spina Christi	100 mg/kg/day oral for 2 weeks	In vivo male albino rats	200 mg/kg/day oral for 2 weeks	↓ ALT, AST, ALP, albumin, and inflammatory factors: IL-1α, IL-1β, IL-4, IL-6, IL-10, IFN-γ, and IFN-α, Inhibition of free radicals	[30]
ACE-Is and ARBs	100 mg,200 mg, 400 mg oral	retrospective analysis, 8-year period, 883 patients	Standard dose	↓Overall APT rate	[31]
Olmesartan Medoxomil	40 mg/kg/day Oral for 4 weeks	In vivo male Sprague Dawley rats	0.6 mg/kg/day, 6 mg/kg/day oral for 4 weeks	↓MDA, lung HP content, and collagen deposition ↑SOD	[129]
*Aronia melanocarpa*	6.25 mg/kg twice, on day 0 and day 2. tracheal injection	In vivo male Wistar rats	5 mL/kg,10 mL/kg day 1 to day 2, day 1 to day 4. oral	↓IL-6, IL-10, number of PMNs in BALF, and pulmonary fibrosis	[32]
Artemisinin	In vitro: 1, 3, and 9 μM incubated with it for 24 h In vivo: Single dose 3 mg/kg, tracheal injection	In vitro: BEAS-2B cell line In vivo: C57Bl6/J male mice	In vitro:6.25 to 100 μM 2 h before exposure to amiodarone In vivo: Single dose of 10 mg/kg, tracheal injection	Downstream Effectors: Nrf2 and SOD1, Upstream Regulator: CaMKK2, Inhibition apoptosis	[33]
Catechin and Epicatechin	concentration of 100 μM	in vitro human lung fibroblast cell line (MRC-5)	10 μM, 100 μM, and 500 μM for 30 min	↑catalase, and SOD, ↓Mitochondrial dysfunction, and cell death	[34]
Curcumin	6.25 mg/kg on day 0 and day 2. tracheal injection	In vivo male Fischer 344 rats	200 mg/kg/day oral for 5 weeks	↓TGF-β1 and c-Jun protein expression, oxidative stress, and pulmonary fibrosis	[35]
Curcumin	40 mg/kg/day oral for 6 weeks	In vivo adult male albino rats	200 mg/kg/day oral for 6 weeks	↓Total leukocyte counts in BALF, IL-6, MDA, and pulmonary fibrosis ↑catalase, and SOD	[36]
Ferulic Acid	30 mg/kg/day oral for 6 weeks	In vivo male albino rats	100 mg/kg/day oral for 6 weeks	↓MDA, AOPP, TGF-β, IL-1β, IL-6, TNF-α. and DNA Damage	[37]
Gallic acid	30 mg/kg/day oral for 6 weeks	In vivo male albino rats	200 mg/kg/day oral for 6 weeks	↑ antioxidant enzymes, ↓Oxidative damage markers, and DNA Damage	[37]
Green Tea	30 mg/kg/day oral for 2 weeks	In vivo adult male albino rat	150 mg/kg/day oral for 2 weeks	↓lung tissue damage	[38]
Human Placenta	100 mg/kg/day IP for 10 days	In vivo Sprague-Dawley male rats	500 μl/kg/day IP for 10 days	Prevention of alveolar septal thickening and volumetric expansion	[39]
l-carnitine	1–5 μM In 24 h exposure	In vitro human lung epithelial cells A549	1,3,10 mM in 24 h exposure	Attenuated mitochondrial membrane depolarization, prevention of cellular ATP depletion, and reduction of PI uptake without altering Annexin-V positivity	[130]
l-carnitine	30 mg/kg/day oral for 60 days	In vivo Wistar albino rats	100 mg/kg/day oral for 30 days after 60 days of exposure to AMD	↓ TNF-α, partially reversed amiodarone-induced alterations in lung tissue	[40]
Nicorandil	60 mg/kg/day oral for 10 weeks	In vivo Wistar albino rats	10 mg/kg/day oral for 10 weeks	↓lung injury and fibrosis observed in histology, oxidative stress, and TGF-β1 expression, Activation of PI3K/Akt1-p/mTOR signaling inhibition	[41]
Paricalcitol	40 mg/kg/day oral for four weeks	In vivo Male Wistar rats	0.2 μg/kg/day IP for four weeks 1 h before amiodarone administration	↑Antioxidants (TAC and GSH) ↓MDA, TLR4 and TNF-α, TGF-\beta1 and pSmad 3 concentrations	[42]
Pirfenidone	A single intratracheal dose of AM, 1.83 μmol	In vivo Male golden Syrian hamsters	0.5% w/w in the chow, early treatment: 3 days before AM instillation, late treatment: started 7 days after AM and continued until sacrifice.	Early treatment: reduced both acute lung injury and subsequent fibrosis. Late treatment: less effective	[43]
Quercetin	6.25 mg/kg on days 0 and 2, intratracheal	In vivo female Wistar rats	20 mg/kg/day oral for 3 weeks	↓Inflammation (TNF-α, IL-1β) and fibrosis, MDA, ↑antioxidant enzymes like SOD and catalase	[44]
Vitamin E	50,100 μM single intratracheal dose	In vitro freshly isolated hamster lung cells	300 μM dietary supplement for 6 weeks	Cell-type specific protection in alveolar macrophages. Did not change the cellular levels of AM	[45]
Vitamin E	In Vivo: A single dose of 1.83 μmol intratracheal instillation In vitro: concentrations 50 μM to 400 μM direct addition of AMD to isolated lung mitochondria	In vivo: male golden Syrian hamster In vitro: freshly isolated hamster lung cells	In vivo: 500 IU of chow dietary supplement for 6 weeks before treatment with AM	Suppression of profibrotic gene expression, ↓overexpression of TGF-β1, and no effect on baseline mitochondrial function	[46]
Vitamin E	concentration range of 10-50 μM, direct incubation for 18 h	In vitro: human pulmonary artery endothelial cells	Concentrations of 10 μM, 20 μM, and 40 μM direct incubation for 18 h	Vitamin E (40 μM) protected the cells from amiodarone toxicity.	[131]
Vitamin E	a single dose of 1.83 mmol transoral intratracheal instillation	In vivo: male golden Syrian hamster	500 IU of chow dietary supplement for 6 weeks before treatment with AM	Prevention of lung fibrosis, Protection against morphological deterioration of the lung, A 234% increase in the vitamin E content of the lung tissue	[132]
Vitamin E	A daily oral dose of 5.4 mg per 100 g of rat body weight for 2 weeks.	In vivo: male albino rats	5 mg/kg twice oral for 2 weeks	↓MDA, improved histological structure, Diffuse positive response in the alveolar lining cells	[47]
cinnamon	50 mg/kg day oral for 10 days	In vivo: male albino Wistar rats	100 mg/kg/day 1 h before AMD for 10 days	↓ MDA, TNF-α, IL-1β, and IL-6 levels in tissue. ↓ NF-κB levels. ↓Creatinine, BUN, and KIM-↑antioxidant enzymes like SOD, catalase, and GSH	[48]
Coenzyme Q10	50 mg/kg day oral for 10 days	In vivo: male albino Wistar rats	10 mg/kg/day 1 h before AMD for 10 days	↓ Oxidative stress (MDA), NF-κB expression, pro-inflammatory cytokines (TNF-α, IL-1β, IL-6), along with creatinine, BUN, and KIM-1 ↑ antioxidants (SOD, GSH, and catalase),	[48]
Grapefruit Juice	18 mg/kg/day oral for 5 weeks	In vivo: adult male Wistar rats	27 ml/kg/day oral for 5 weeks	↓Amount of fragmented DNA, ↓creatinine, urea, ↑glycogen, and total protein contents	[49]
Grape Seed	30 mg/kg/day oral for 8 weeks	In vivo: male albino rats	100 mg/kg/day oral for 8 weeks	↓MDA, IL-6, and TNF-α, ↑SOD	[50]
Zizyphus Spina Christi	100 mg/kg/day oral for 2 weeks	In vivo: adult male albino rats	200 mg/kg day oral for 2 weeks	↓Creatinine, urea, and Inflammatory factor: IL-1α, IL-1β, IL-4, IL-6, IL-10, IFN-γ, and IFN-α, Inhibition free radical	[30]
Mesenchymal stem cells	100 mg/kg/day orally for 4 weeks	In vivo: adult albino Wistar rats	1 × 10^^6^ single dose cells/animal via tail-vein injection	Restoration of thyroid hormone levels to normal, ↑SOD and GPx, ↓MDA, by upregulation of Bcl-2, downregulation of BAX, Caspase-3, IL-6, VEGF, and iNOS.	[51]
Mesenchymal stem cells with melatonin	100 mg/kg/day orally for 4 weeks	In vivo: adult albino Wistar rats	50 mg/kg single dose intravenously	↑T3 and T4, ↓ TSH, oxidative stress, apoptosis, and fibrosis,	[51]
Gilaburu	100 mg/kg/day IP for 10 days	In vivo: male Wistar rats	100 mg/kg/day oral for 10 days	↓Histopathological alterations and TNF-α expression	[52]
Grapefruit juice	18 mg/kg/day for 5 weeks	In vivo: adult male albino rats	27 mL/kg/day for 5 weeks	↓Cytotoxicity and testicular alteration	[128]

**Table 2:** Comparative evidence grading and translational readiness.

Intervention/group	Organ	Replication	Mechanistic coherence	Strength	Readiness
Vitamin E	Lung; liver	Multiple reports	Redox/TGF-β/morphology	Moderate preclinical	Low
Curcumin	Lung	Two animal reports	Redox/inflammation/fibrosis	Moderate preclinical	Low
Silymarin	Liver	Two animal reports	GSH/inflammation/phospholipidosis	Moderate preclinical	Low
l-carnitine	Lung	In vitro + animal	Mitochondria/ATP/inflammation	Moderate preclinical	Low
Grape-seed preparations	Liver; kidney	Separate animal reports	Redox/inflammation/structure	Moderate–low	Low
ACE-I/ARB exposure	Lung	One human observational report	RAS/apoptosis hypothesis	Very low	Confounded human signal
Other compounds	Multiple	Single preclinical studies	Mostly downstream redox/inflammation	Low–very low	Very low

Note: “Moderate” denotes replicated preclinical evidence, not demonstrated clinical efficacy. Evidence categories are descriptive and are not a formal GRADE assessment.

### 2.5 Study identification

The search and manual screening identified 89 records. Nine duplicates were removed, leaving 80 records for title/abstract screening. Forty-five records were excluded and 35 full texts were retrieved; all 35 met the eligibility criteria and were included. The low yield reflects the narrow requirement for direct testing of a protective intervention in an amiodarone-toxicity model. Background mechanistic studies and studies of general antioxidant effects were not eligible evidence. No retrieved full-text report was excluded. The study-selection process is shown in Fig. 1.

### 2.6 Risk of bias assessment

Animal intervention studies were assessed with SYRCLE's risk-of-bias tool. In vitro studies were evaluated with an adapted OHAT framework covering exposure characterization, allocation, blinding, outcome validity, incomplete data, selective reporting, and conflicts of interest. The human observational study was assessed using the applicable JBI checklist. Two reviewers judged each study independently and resolved disagreements by consensus. Risk-of-bias findings informed interpretation: evidence from studies with predominantly unclear or high-risk domains was not treated as confirmatory, even when biomarker effects were statistically significant. Design-specific risk-of-bias assessments are summarized in the supplementary risk-of-bias table.

For animal studies, SYRCLE domains included sequence generation, baseline characteristics, allocation concealment, random housing, blinding of investigators/caregivers, random and blinded outcome assessment, incomplete data, selective reporting, and other bias. Judgments were low, high, or unclear risk. The original figure indicates substantial unclear reporting, particularly for allocation and blinding; this limitation is integrated into the Discussion.

### 2.7 Assessment of reporting bias

Because few studies evaluated the same intervention–outcome combination and no quantitative synthesis was performed, funnel plots and statistical tests for small-study effects were not considered appropriate. The potential for reporting bias was considered qualitatively based on the predominance of small, positive preclinical studies. No formal quantitative assessment of reporting bias was performed.

## 3 Active ingredients against amiodarone-induced hepatotoxicity

### 3.1 Adenosine triphosphate

Adenosine triphosphate (ATP) is a crucial molecule recognized for its vital role as an intracellular energy source and in nucleic acid biosynthesis. It also contributes to the synthesis of antioxidants that neutralize reactive oxygen species (ROS), providing the energy needed for these processes. In an animal study, Adenosine triphosphate (ATP) has been shown to protect against amiodarone-induced hepatotoxicity by reducing oxidative stress and restoring antioxidant defenses. Amiodarone administration is linked to increased ROS production, mitochondrial dysfunction, ATP depletion, and subsequent liver cell injury. Exogenous ATP supplementation has been demonstrated to decrease oxidative stress markers, such as malondialdehyde (MDA), while restoring antioxidant levels, including total glutathione (tGSH), superoxide dismutase (SOD), and catalase (CAT). Moreover, ATP treatment lessened serum levels of alanine aminotransferase (ALT) and aspartate aminotransferase (AST), indicating improved liver function. Histopathological analysis showed reduced vascular dilation, lipid accumulation, necrosis, and inflammatory cell infiltration in groups treated with ATP. Immunohistochemical results further revealed that ATP downregulated caspase-3 and tumor necrosis factor-alpha (TNF-α), thereby decreasing apoptosis and inflammation, which supports its hepatoprotective potential [20].

### 3.2 Chard (*Beta vulgaris L. var. cicla*)

Chard (*Beta vulgaris* L. var. cicla) is a widely recognized plant that has been traditionally used as a folk remedy for liver and kidney disorders, as well as for enhancing immune function and preventing cancer [53]. Compared to spinach and celery, it is relatively easy to cultivate, making it a readily accessible dietary component. Its leaves and stems are commonly consumed as vegetables and are valued for both their nutritional and therapeutic properties. Numerous studies have highlighted its diverse biological activities, including antioxidant, anti-acetylcholinesterase, antidiabetic, and antithrombotic effects, underscoring its potential role in promoting human health and preventing disease [54,55]. A preclinical study evaluated the hepatoprotective effects of chard against AMD-induced liver injury. AMD administration resulted in significant hepatic injury, as indicated by elevated serum and hepatic biomarkers, including total lipids, cholesterol, bilirubin, lipid peroxidation (LPO), and liver enzymes (AST, ALP, LDH), along with a reduction in GSH levels. Histopathological analysis revealed necrosis and inflammatory infiltration in hepatic tissue. Co-treatment with chard extracts significantly ameliorated these biochemical and histological alterations. The hepatoprotective effects were attributed to the antioxidant activity of chard, likely due to its high content of flavonoids and phenolic compounds. These findings highlight the potential of chard extract as a preclinical candidate for attenuating AMD-induced hepatotoxicity [21].

### 3.3 Diacerein

Diacerein (DcN), an anthraquinone derivative primarily indicated for osteoarthritis and other inflammatory conditions such as periodontitis, psoriasis, and epidermolysis bullosa, exerts its pharmacological effects through its active metabolite, rhein, which possesses anti-inflammatory, analgesic, and antipyretic properties [56]. Functioning as an interleukin inhibitor, DcN suppresses key pro-inflammatory cytokines, including IL-1β, IL-6, and TNF-α, thereby modulating inflammatory responses and tissue degeneration. Beyond its established clinical uses, DcN has demonstrated significant hepatoprotective potential against amiodarone AMD-induced hepatic injury. AMD-mediated hepatotoxicity, characterized by oxidative stress, inflammation, and apoptosis through activation of the TLR4/NF-κB/NLRP3 signaling pathway, was markedly attenuated by DcN treatment. DcN improved liver function and histological integrity, enhanced antioxidant capacity via upregulation of Nrf2 and GSH, and reduced lipid peroxidation markers such as MDA. Furthermore, DcN downregulated inflammatory mediators (TLR4, NF-κB p65, NLRP3, Caspase-1, IL-1β) and suppressed Caspase-3 expression, thereby inhibiting apoptosis. Collectively, these findings highlight DcN as a promising candidate for further preclinical evaluation with integrated antioxidant, anti-inflammatory, and anti-apoptotic mechanisms, offering protection against drug-induced hepatic injury [57].

### 3.4 Ginkgo biloba

*Ginkgo biloba*, a medicinal plant widely used in traditional and modern medicine, is recognized for its potent antioxidant activity, primarily due to its flavonoid and terpenoid content. These compounds neutralize reactive oxygen species, limit lipid peroxidation, and protect cells from oxidative stress. Experimental and clinical evidence highlight its neuroprotective, hepatoprotective, and cardioprotective effects. Its preclinical potential is primarily attributed to antioxidant mechanisms that preserve cellular integrity and function [22,58]. AMD-induced hepatotoxicity in rats was ameliorated through improvements in biochemical, genetic, and structural alterations of the liver. Co-administration significantly reduced serum ALT and AST elevations, alleviated oxidative stress, and decreased DNA fragmentation as demonstrated by the comet assay. Histopathological evaluation showed marked protection against fatty degeneration, inflammatory cell infiltration, sinusoidal dilation, and collagen deposition, while histochemical analysis revealed partial restoration of hepatic glycogen content. Ultrastructural examination further confirmed improved cellular integrity, with preservation of nuclei, mitochondria, and endoplasmic reticulum. The hepatoprotective action of *Ginkgo biloba* is mediated mainly by its antioxidant capacity and ability to counteract amiodarone-induced cellular injury [59].

### 3.5 Glycyrrhiza glabra

*Glycyrrhiza glabra* (licorice) has been used in traditional medicine for thousands of years to treat hepatic and gastrointestinal disorders, respiratory conditions, and inflammatory diseases [23]. The roots of the plant are pharmacologically significant owing to their abundance of bioactive compounds, most notably glycyrrhizin, a triterpenoid saponin that is approximately 50 times sweeter than sucrose and possesses diverse therapeutic properties. Extensive evidence has demonstrated that *G. glabra* exhibits anti-inflammatory, antioxidant, anti-ulcer, antimalarial, expectorant, antispasmodic, diuretic, laxative, and sedative activities, underscoring its broad pharmacological potential and relevance as a candidate for hepatoprotective interventions [60,61]. Oral pretreatment with graded doses of *Glycyrrhiza glabra* significantly normalized serum GSH, SOD, and MDA levels, thereby conferring protection against oxidative stress and histopathological liver injury, with the most pronounced effect observed at the highest dose. The hepatoprotective activity was primarily mediated through enhancement of antioxidant capacity, suppression of lipid peroxidation, and modulation of inflammatory pathways, underscoring its potential as a candidate intervention in experimental amiodarone-induced hepatotoxicity [62].

### 3.6 Grape seed

Grape seed extract, rich in procyanidins, demonstrates potent antioxidant activity by neutralizing reactive oxygen species, inhibiting apoptosis, and supporting DNA repair mechanisms [24]. Its hepatoprotective potential has been evidenced through reduced serum ALT and AST levels, improved hepatocyte function, and enhanced glycogen storage in the liver and muscle [63]. In an amiodarone-induced hepatotoxicity model, grape seed extract significantly ameliorated biochemical disturbances, DNA damage, and structural alterations. Specifically, co-administration with amiodarone reduced liver enzyme elevations, decreased comet assay indices of DNA damage, and restored a greater proportion of undamaged hepatocytes. Histopathological and histochemical analyses further revealed protection against fatty degeneration, inflammatory infiltration, sinusoidal dilation, collagen fiber deposition, and glycogen depletion. Meanwhile, ultrastructural evaluation confirmed the preservation of nuclei, mitochondria, and endoplasmic reticulum. Collectively, these findings indicate hepatoprotective activity in the tested model of grape seed extract, which is likely mediated by its antioxidant properties and reduction of lysosomal phospholipidosis.

### 3.7 Livogrit

Livogrit is a tri-herbal Ayurvedic medicine formulated with *Boerhavia diffusa* (Punarnava), *Phyllanthus niruri* (Bhumi amalaki), and *Solanum nigrum* (Makoy), containing a blend of antioxidant-rich phytochemicals such as quercetin, rutin, gallic acid, and catechin, and traditionally recognized for its hepatoprotective, lipid-lowering, and anti-inflammatory properties.

Livogrit demonstrated significant hepatoprotective activity both in vitro (HepG2 cells) and in vivo (*Caenorhabditis elegans*) models of amiodarone-induced hepatotoxicity. The intervention exerted its effects through the restoration of lysosomal acidity, attenuation of phospholipid accumulation, and reduction of oxidative stress by enhancing the activity of antioxidant enzymes (SOD, catalase, GSH, GST). Moreover, Livogrit normalized elevated hepatic biomarkers (AST, ALT, and cholesterol), downregulated lipid biosynthesis genes (SCD-1, LSS), preserved lysosomal phospholipase A2 expression, and reinstated P-glycoprotein function, thereby reducing intracellular amiodarone accumulation. Collectively, these findings suggest that Livogrit confers hepatoprotection through the modulation of redox homeostasis, lipid metabolism, and cellular efflux mechanisms [64].

### 3.8 *Mangifera indica* L. Stem bark

The aqueous stem bark extract of *Mangifera indica* L. (MSBE), traditionally used in Cuban medicine for its restorative and hepatoprotective properties, is a polyphenol-rich natural preparation composed of terpenoids, steroids, fatty acids, and trace elements, with mangiferin identified as its main bioactive component (around 20% of the extract) [25,65]. Evidence from in vitro studies using HepG2 human hepatocytes shows that both MSBE and mangiferin provide significant protection against AMD-induced mitochondrial and oxidative damage. Amiodarone is known to impair mitochondrial β-oxidation and oxidative phosphorylation, leading to membrane depolarization, calcium imbalance, excessive reactive oxygen species (ROS) production, and lipid peroxidation, which ultimately decreases cell viability. MSBE and mangiferin reduce these toxic effects through multiple, interconnected mechanisms, including direct scavenging of ROS and other free radicals, stabilization of mitochondrial membrane potential, preservation of ATP production, regulation of intracellular Ca^2+^ levels, and boosting natural antioxidant defenses such as SOD, catalase, and GSH activity. Pre-treatment with MSBE or mangiferin offers better protection than co-treatment, indicating a preconditioning effect that strengthens cellular antioxidant capacity before toxic exposure. Additionally, the whole extract shows comparable or even better cytoprotective effects than isolated mangiferin, suggesting synergistic interactions among its polyphenolic components. Overall, these findings support the potential of MSBE as a multi-target hepatoprotective agent that can counteract xenobiotic-induced mitochondrial dysfunction through antioxidant, mitochondrial stabilization, and calcium-regulation mechanisms [66].

### 3.9 Melatonin

Melatonin (MLT), a neurohormone predominantly secreted by the pineal gland during darkness, regulates circadian and neuroendocrine rhythms via MT1 and MT2 receptors [26,67]. Beyond its chronobiotic function, MLT possesses antioxidant, anti-inflammatory, and antiapoptotic properties that mitigate oxidative stress and cellular injury, and it has also been implicated in anticancer activity through modulation of tumor progression, angiogenesis, and cellular differentiation [68]. In experimental models, coadministration of MLT with amiodarone (AMD) significantly attenuated AMD-induced hepatotoxicity, as evidenced by reductions in serum liver enzymes (ALT, AST, LDH, γ-GT), oxidative stress markers (xanthine oxidase activity, TBARS, protein carbonyl content, and p53 expression), and the severity of histopathological alterations, including hepatocyte degeneration, microvesicular steatosis, and focal necrosis [69].

### 3.10 Resveratrol

Resveratrol (RV) is a widely studied polyphenolic compound present in various plant sources, including grapes, peanuts, and berries, and is recognized for its broad spectrum of health-promoting effects. Evidence from preclinical and clinical studies highlights the anti-obesity, cardioprotective, neuroprotective, antitumor, antidiabetic, antioxidant, and anti-aging properties of this compound, as well as its role in regulating glucose metabolism. Notably, resveratrol has demonstrated promising preclinical potential in cancer, neurodegenerative disorders, and atherosclerosis, primarily through the modulation of oxidative stress, apoptosis, and inflammatory pathways [27,70,71]. Concomitant administration of resveratrol demonstrated a substantial protective effect against AMD-induced hepatotoxicity. Specifically, resveratrol significantly reduced lipid peroxidation, as indicated by decreased MDA levels, and enhanced the endogenous antioxidant system by restoring GSH and SOD levels. It also lowered serum AST activity, reflecting improved hepatocellular function, and ameliorated histopathological alterations such as hepatocyte degeneration and necrosis, as well as immunohistochemical indicators of apoptosis and inflammation (caspase-3 and TNF-α). However, its effects on ALT activity and catalase levels were comparatively limited, suggesting an incomplete restoration of liver function. Of course, Concurrent treatment with resveratrol and ATP demonstrated synergistic hepatoprotective effects by normalizing biochemical markers and attenuating oxidative stress [20].

### 3.11 Silymarin

Silymarin (SLM) is a flavonoid-type antioxidant recognized for anti-proliferative, anti-fibrotic, anti-apoptotic, antiviral, and immunomodulatory effects. Hepatoprotective effects of silymarin against galactosamine, thioacetamide, halothane, and carbon tetrachloride poisoning have been proven [72,73]. In two independent investigations, AMD caused widespread hepatic histological and ultrastructural alterations, including vacuolation, leucocytic infiltration, mitochondrial degeneration, nuclear pyknosis, and increased lysosomal phospholipidosis with characteristic myelin figures. Also, AMD exposure elevated liver injury enzymes (ALT, AST), oxidative stress markers (malondialdehyde, conjugated dienes, thiobarbituric acid reactive substances), and inflammatory cytokines (TNF-α, IL-6). SLM treatment resulted in improved liver histology and ultrastructure, reduced levels of liver enzymes, and decreased markers of oxidative stress and inflammation. Silymarin's hepatoprotective efficacy is attributed to its potent antioxidant and free radical scavenging properties, which regulate intracellular glutathione content and stimulate liver regeneration. Additionally, one study suggests a reduction in hepatic amiodarone concentrations. These findings underscore silymarin's consistent defensive role against AMD-induced liver damage [29,74].

### 3.12 Vitamin E

Vitamin E, a lipid-soluble vitamin comprising tocopherols and tocotrienols, is recognized for both antioxidant and anti-inflammatory activities. These properties contribute to roles in neuroprotection, cardiovascular regulation, and the maintenance of skin and bone health [28]. In the context of AMD-induced hepatotoxicity, vitamin E supplementation significantly reduced hepatic oxidative stress markers, including conjugated dienes and TBARS. It also decreased lysosomal phospholipidosis, reducing both the number and size of pathological lysosomes. These protective effects occurred without altering hepatic amiodarone concentrations, indicating independence from drug accumulation. Overall, evidence supports vitamin E as a modulator of oxidative injury and hepatocellular damage in drug-induced liver toxicity [74].

### 3.13 Zizyphus spina christi

Zizyphus spina-christi (ZSC) is traditionally recognized for its soothing, wound-healing, antiseptic, anti-inflammatory, and antimicrobial properties, and is also used to treat digestive, urinary, and metabolic disorders [75]. Its preclinical potential arises from a rich phytochemical composition, including flavonoids, tannins, sterols, betulinic acid, peptide alkaloids, and triterpenoidal saponin glycosides [76]. Research across multiple hepatotoxicity models, such as chemical toxins, heavy metals, infections, and drug-induced damage, demonstrates consistent hepatoprotective activity [77,78]. These benefits are associated with improvements in biochemical markers, including ALT, AST, ALP, and albumin, as well as the suppression of inflammatory mediators and the preservation of liver histology. Amiodarone (AMD), a drug known for causing hepatotoxicity, leads to elevated liver enzymes, reduced albumin, and histopathological damage, including vacuolated hepatocytes and vascular congestion. Co-administration of ZSC extract has been shown to counteract these effects by restoring enzyme levels, maintaining tissue integrity, and reducing inflammation. This protective action is mainly attributed to its antioxidant role in scavenging free radicals and its anti-inflammatory capacity to downregulate cytokines and interferons, positioning ZSC as a promising natural hepatoprotective agent against drug-induced liver injury [79].

## 4 Active ingredients against amiodarone-induced pulmonary toxicity

### 4.1 Angiotensin-converting enzyme inhibitors (ACE-is) and angiotensin receptor blockers (ARBs)

The renin-angiotensin system (RAS) plays a crucial role in regulating blood pressure, fluid balance, and pulmonary function, with angiotensin II being a key driver of vasoconstriction, fibrosis, and the progression of lung disease. Dysregulation of this pathway is implicated in chronic respiratory disorders such as pulmonary hypertension and fibrosis, as well as acute conditions like acute respiratory distress syndrome (ARDS) [30]. Pharmacological inhibition of RAS using angiotensin-converting enzyme inhibitors (ACE-Is) and angiotensin receptor blockers (ARBs) has demonstrated protective effects against amiodarone-induced pulmonary toxicity (APT). Clinical studies show that patients treated with ACE-Is or ARBs have a significantly lower incidence of APT compared with untreated individuals. Preclinical models support these findings, with olmesartan, an AT1 receptor antagonist, demonstrating a reduction in fibrosis, oxidative stress, and profibrotic signaling. These benefits are associated with the suppression of transforming growth factor-β1 and type I collagen expression, as well as enhanced antioxidant activity. Collectively, evidence highlights RAS blockade as a promising therapeutic approach to attenuate amiodarone-induced pulmonary damage through anti-apoptotic, anti-inflammatory, and antifibrotic mechanisms [80,81].

### 4.2 Aronia melanocarpa

Black chokeberry (*Aronia melanocarpa*) is a lesser-known fruit commonly processed into juices, jams, and dietary supplements, and is recognized for its high antioxidant content, particularly phenolic acids and flavonoids [31]. Evidence from the scientific literature indicates that *A. melanocarpa* supports physiological homeostasis by mitigating oxidative stress and inflammation, while emerging studies also suggest beneficial effects on intestinal health. These properties are of particular relevance given the interconnection between oxidative stress, inflammation, the gut microbiome, and overall well-being [31]. Preclinical research has further demonstrated that *A. melanocarpa* fruit juice exerts protective effects against amiodarone-induced pulmonary toxicity, as evidenced by improvements in lung tissue parameters, reductions in oxidative stress markers and fibrosis, and modulation of inflammatory mediators. Notably, treatment restored levels of the anti-inflammatory cytokine interleukin-10 and suppressed the expression of the pro-inflammatory cytokine interleukin-6. Collectively, these findings highlight the potential of *A. melanocarpa* as a functional food with significant antioxidant and anti-inflammatory properties, which are relevant to systemic health [82].

### 4.3 Artemisinin

Artemisinin (ART), also known as Qinghaosu, is a natural compound initially extracted from *Artemisia annua* and widely recognized for its potent antimalarial and antitumor activities, the latter partly attributed to inducing oxidative stress [32]. However, its pharmacological profile also encompasses significant cytoprotective properties, including anti-inflammatory, antifibrotic, antioxidative stress, and antiapoptotic effects [83]. Recent research indicates that artemisinin effectively protects human bronchial epithelial cells and lung tissue from amiodarone-induced oxidative damage and apoptosis. This protective mechanism is critically dependent on the activation of the 5′-AMP-activated protein kinase (AMPK) signaling pathway, specifically via upstream CaMKK2 kinase rather than LKB1. This activation subsequently upregulates critical antioxidant defense proteins like Nrf2 and SOD1, thereby mitigating cellular injury [84].

### 4.4 Catechin and epicatechin

Catechin and epicatechin are naturally occurring flavan-3-ol polyphenols widely distributed in plant-derived foods and beverages (e.g., green tea, cocoa, apples, and berries) and are characterized by pronounced antioxidant and cytoprotective activities [33]. As stereoisomers, their distinct stereochemical configurations may influence pharmacokinetic behavior and biological potency, including the capacity to scavenge reactive oxygen species, chelate redox-active metals, and modulate redox-sensitive signaling pathways [85]. In experimental settings, both compounds have been reported to attenuate amiodarone (AMD)–induced cytotoxicity in human lung fibroblasts (MRC-5) by counteracting mitochondrial complex I impairment, improving ATP generation, reducing lipid and protein oxidative damage, restoring antioxidant enzyme activity, maintaining nitric oxide levels, and ultimately preserving cell viability [86]. Concordantly, preclinical animal studies suggest that catechin-rich green tea (*Camellia sinensis*) extract, particularly epigallocatechin-3-gallate (EGCG), attenuates AMD associated pulmonary histopathological alterations, including alveolar collapse, pneumocyte degeneration, septal thickening, collagen deposition, and inflammatory cell infiltration [34]. Collectively, the available preclinical evidence supports a potential mechanistic role for catechin-based interventions in mitigating AMD-related pulmonary injury, predominantly through mitochondrial preservation and redox homeostasis.

### 4.5 Curcumin

Curcumin, a lipophilic compound derived from the rhizomes of *Curcuma longa* L., has been extensively investigated for its diverse pharmacological activities. Evidence indicates that it exerts antiviral, antimicrobial, and antitumor effects, in addition to modulating lipid and glucose metabolism. More recently, its antifibrotic potential has been demonstrated in rat models of bleomycin-induced lung fibrosis. curcumin supplementation attenuated amiodarone-induced pulmonary fibrosis by reducing oxidative stress markers, suppressing pro-fibrotic mediators such as transforming growth factor-β1 (TGF-β1), and downregulating c-Jun expression, thereby limiting structural lung damage and collagen deposition [87]. More recent comparative studies further corroborate these findings, demonstrating that curcumin not only mitigates histopathological and biochemical markers of lung injury but also provides superior protection relative to other antioxidants, such as melatonin. Collectively, these results suggest that curcumin holds preclinical potential as an adjunctive strategy to ameliorate ATP; however, translation into clinical practice requires validation in well-designed human trials [35].

### 4.6 Ferulic acid

Ferulic acid (FA), a naturally occurring phenolic compound, exhibits diverse pharmacological activities, including antioxidant, anti-inflammatory, antidiabetic, anticancer, and immunomodulatory effects [36]. Its therapeutic efficacy is primarily attributed to its capacity to scavenge reactive oxygen species, suppress pro-inflammatory cytokines, and protect biomolecules such as DNA and lipids from oxidative damage [88]. Evidence indicates that FA administration alleviates oxidative stress, reduces DNA injury, and attenuates histopathological alterations, thereby preserving pulmonary architecture and function during amiodarone-induced toxicity [89]. Additionally, FA enhances the activity of antioxidant enzymes, reduces lipid peroxidation, and supports the maintenance of cellular redox homeostasis. Collectively, these findings underscore the pharmacological relevance of FA as a protective agent against oxidative and inflammatory injury associated with drug-induced organ toxicity.

### 4.7 Gallic acid

Gallic acid (GA), a naturally occurring phenolic compound and potent antioxidant derived from plant polyphenols such as gallotannins, is broadly distributed in natural sources, including tea leaves, grapes, berries, apples, and gallnuts [90]. It exhibits diverse biological activities—antimicrobial, anticancer, anti-ulcer, anti-hyperglycemic, neuroprotective, and lipid-regulating—primarily attributable to its strong antioxidant potential [91]. In models of amiodarone-induced pulmonary toxicity, GA has demonstrated significant protective efficacy by attenuating oxidative stress, preventing DNA damage, and alleviating histopathological alterations, thereby preserving lung architecture and function. Furthermore, GA enhances antioxidant enzyme activity, suppresses inflammatory biomarkers such as TNF-α and IL-6, and reduces inflammatory cell infiltration, underscoring its pronounced anti-inflammatory properties. It has also been shown to restore redox balance, mitigate fibrotic changes, and modulate apoptotic pathways, collectively highlighting its ability to protect tissues from oxidative and inflammatory injury. Taken together, these findings position GA as a promising therapeutic candidate for mitigating drug-induced pulmonary and systemic toxicities [92].

### 4.8 Green tea

Green tea (*Camellia sinensis*) is recognized for its broad spectrum of pharmacological effects, attributed mainly to its abundant catechins, particularly (−)-epigallocatechin-3-gallate (EGCG), as well as caffeine and amino acids. These constituents exert antioxidant, anticancer, cardioprotective, anti-diabetic, anti-obesity, neuroprotective, antimicrobial, and photoprotective effects through the modulation of key signaling pathways and the reduction of oxidative stress. Experimental findings highlight its protective role against drug-induced organ toxicity, as evidenced by the ability of green tea extract to alleviate APT in animal models. Amiodarone exposure typically results in structural damage characterized by alveolar collapse, pneumocyte degeneration, septal thickening, collagen accumulation, and macrophage infiltration. Co-administration of green tea extract markedly reduced these pathological alterations, preserving lung architecture and function. The observed protective effect is attributed to the potent antioxidant properties of catechins, which counteract oxidative stress-mediated tissue injury [37].

### 4.9 Human placental

Human Placenta Extract (HPE), a biologically active substance enriched with growth factors, hormones, proteins, amino acids, enzymes, cytokines, vitamins, and minerals, has long been utilized in Asian countries for therapeutic purposes due to its antioxidant and anti-inflammatory properties [38,93]. Findings demonstrated that amiodarone administration markedly increased alveolar septal volume and thickness, while significantly reducing alveolar space and volume, along with causing red blood cell accumulation and septal inflammation. Concurrent treatment with HPE effectively ameliorated these pathological alterations, thereby preserving lung architecture. The protective effects of HPE are attributed to its antioxidant and anti-inflammatory actions, collagen content, and its ability to promote tissue regeneration and cellular proliferation. Overall, these results indicate that HPE protects against amiodarone-induced structural lung damage [94].

### 4.10 l-carnitine

l-carnitine (LC) is an endogenously synthesized compound that supports energy production by transporting fatty acids into mitochondria. It enhances myocardial metabolism, improves exercise tolerance, and is therapeutically used in primary deficiency and renal disease. Additionally, it exhibits antioxidant and anti-inflammatory effects, with potential roles in lipid regulation and neuroprotection; however, evidence remains limited. Evidence also supports its protective role against amiodarone-induced pulmonary toxicity. In rat models, amiodarone caused oxidative stress, inflammation, and fibrosis, marked by elevated MDA and tumor necrosis factor-alpha (TNF-α) with reduced CAT, SOD, and reduced GSH, while LC co-administration ameliorated these alterations and partially restored antioxidant defenses, but TNF-α levels were significantly reduced [39]. In vitro studies on A549 lung epithelial cells have demonstrated that amiodarone-induced cytotoxicity involves mitochondrial depolarization, ATP depletion, and the activation of both necrotic and apoptotic pathways. Treatment with LC reduced mitochondrial dysfunction and necrosis, though apoptotic markers were unaffected. These findings suggest LC exerts protection through antioxidant and mitochondrial-stabilizing mechanisms. However, targeting both necrosis and apoptosis may be required for more effective prevention of drug-induced lung injury [95].

### 4.11 Nicorandil

Nicorandil (N-[2-hydroxyethyl]-nicotinamide nitrate) is an established anti-anginal agent that exerts dual pharmacological actions as a nitric oxide donor and potassium channel opener, thereby facilitating bronchodilation and enhancing oxygen delivery [40]. Beyond its cardiovascular utility, nicorandil exhibits pronounced antioxidant, anti-inflammatory, and antifibrotic activities, primarily through mechanisms involving free radical scavenging, downregulation of iNOS, and upregulation of eNOS. Growing evidence suggests that nicorandil offers significant protection against pulmonary toxicity induced by agents such as bleomycin, silica, and cyclophosphamide [96,97]. In the context of amiodarone-induced pulmonary toxicity, nicorandil has been shown to attenuate respiratory impairment, histopathological alterations, collagen accumulation, and oxidative stress, while suppressing activation of the TGF-β1/PI3K/Akt/mTOR signaling cascade, a central mediator of fibrogenesis. It further reduces fibroblast proliferation and the expression of fibrosis-associated markers, while restoring the eNOS/iNOS balance, thereby reinforcing its antioxidant and anti-inflammatory profile. Collectively, these findings suggest that nicorandil represents a promising therapeutic candidate for the prevention and mitigation of drug-induced pulmonary injury through modulation of oxidative stress and profibrotic signaling pathway [98].

### 4.12 Paricalcitol

Paricalcitol, a synthetic analog of calcitriol and a selective vitamin D receptor (VDR) activator marketed as Zemplar®, is commonly used to treat secondary hyperparathyroidism related to chronic kidney disease. Emerging evidence indicates that, beyond its renal applications, paricalcitol has notable cytoprotective effects in pulmonary injury through its anti-inflammatory, antioxidant, and antifibrotic actions [41,99]. In AMD-induced lung toxicity, characterized by oxidative stress, inflammatory infiltration, and fibrotic remodeling, paricalcitol significantly alleviated pathological changes by downregulating TLR4, NF-κB p65, and TNF-α expression, restoring redox balance through increased GSH and total antioxidant capacity (TAC), and decreasing MDA levels. Furthermore, it attenuated fibrogenesis through inhibition of the TGF-β1/pSmad3 signaling pathway, decreased α-SMA expression and collagen deposition, and suppressed hypoxia-inducible factor-1α (HIF-1α), a critical mediator linking inflammation and fibrosis. Collectively, these findings support paricalcitol potential as a therapeutic agent that mitigates amiodarone-induced pulmonary toxicity through integrated antioxidant, anti-inflammatory, and antifibrotic mechanisms [100].

### 4.13 Pirfenidone

Pirfenidone is an oral antifibrotic and anti-inflammatory drug approved for idiopathic pulmonary fibrosis (IPF). It primarily acts by inhibiting profibrotic cytokines, such as TGF-β1 and PDGF (Platelet-derived growth factor), thereby reducing fibroblast proliferation, extracellular matrix accumulation, and collagen synthesis. In addition, its antioxidant and anti-inflammatory effects suppress pro-inflammatory mediators, helping to limit tissue injury and fibrotic remodeling [42,101]. Pirfenidone has been shown to effectively prevent amiodarone-induced pulmonary fibrosis when administered prophylactically. In contrast, delayed treatment did not confer protection, indicating that its efficacy is linked to modulation of early pathogenic events. Its antifibrotic action is primarily associated with suppression of TGF-β1 gene expression, a central mediator of extracellular matrix remodeling and collagen deposition. Although pirfenidone did not alleviate amiodarone-induced mitochondrial dysfunction, macrophage death, or acute neutrophilic inflammation, it significantly reduced eosinophilic infiltration, highlighting additional anti-inflammatory properties. These findings suggest that pirfenidone attenuates fibrotic progression predominantly through interference with early pro-fibrotic signaling pathways [102].

### 4.14 Quercetin

Quercetin is a plant-derived flavonoid abundant in fruits, vegetables, and tea, with well-documented antioxidant, anti-inflammatory, antifibrotic, and antiviral activities [103]. It exerts its pharmacological effects through mechanisms such as scavenging reactive oxygen species, enhancing endogenous antioxidant defenses, inhibiting pro-inflammatory cytokines and NF-κB (nuclear factor k-light-chain-enhancer of activated B cells) signaling, and suppressing TGF-β1-mediated collagen deposition [43]. Importantly, quercetin has been shown to have protective effects against amiodarone-induced pulmonary toxicity, a condition primarily driven by oxidative stress and inflammation. In experimental models, quercetin significantly reduced macrophage infiltration in bronchoalveolar lavage fluid and alleviated pulmonary inflammation in rats treated with amiodarone. These protective effects are attributed to its capacity to suppress inflammatory cell recruitment and neutralize reactive oxygen species. Collectively, the evidence highlights quercetin as a promising candidate for mitigating amiodarone-induced lung injury through the modulation of oxidative and inflammatory pathways [104].

### 4.15 Vitamin E

Vitamin E is a lipid-soluble, chain-breaking antioxidant recognized primarily for its free radical scavenging properties. It functions as a key component of the non-enzymatic antioxidant defense system, notably preventing lipid peroxidation and stabilizing cell membranes [105]. Research across multiple studies indicates that vitamin E offers protective effects against amiodarone-induced pulmonary damage in AMD. In vitro, it selectively attenuated AMD-induced cytotoxicity in alveolar macrophages, a protection not linked to altered drug levels or lipid peroxidation. In vivo, vitamin E prevented AMD-induced pulmonary fibrosis in hamsters, reducing hydroxyproline content and histological damage, and suppressing the expression of TGF-β1 mRNA. This protection, however, did not extend to mitigating the mitochondrial dysfunction induced by AMD. Furthermore, in rats, α-tocopherol ameliorated AMD-induced histopathological changes, including improved alveolar integrity, and significantly reduced lipid peroxidation. Collectively, these studies highlight vitamin E role in cell-type selective cytotoxicity reduction, profibrotic gene modulation, and lipid peroxidation reduction, as mechanisms against AMD-induced lung injury [44,45,106,107].

## 5 Active ingredients against amiodarone-induced nephrotoxicity

### 5.1 Cinnamon

Cinnamon (CE), derived from the dried inner bark of Cinnamomum species, has been widely applied in culinary practices, food preservation, and traditional medicine, with its pharmacological effects primarily attributed to bioactive compounds such as cinnamaldehyde, cinnamic acid, and eugenol [46]. These constituents confer potent antioxidant, anti-inflammatory, antimicrobial, and metabolic regulatory activities, supporting their potential in the management of oxidative stress, metabolic dysfunction, and infectious conditions [47,108]. Experimental studies have demonstrated that cinnamon extract exerts significant nephroprotective effects against amiodarone-induced toxicity in rats. These effects are evidenced by reductions in oxidative stress markers (MDA), restoration of antioxidant defenses (GSH, SOD, catalase), suppression of inflammatory mediators (NF-κB, TNF-α, IL-1β, IL-6), and improvements in kidney function indicators (serum creatinine, BUN, KIM-1). Histopathological analyses further confirmed the attenuation of renal tissue injury. Collectively, these findings underscore the potential of cinnamon extract as both a functional food component and a promising therapeutic candidate for the prevention of drug-induced nephrotoxicity [109].

### 5.2 Coenzyme Q10

Coenzyme Q10 (CoQ10), also known as ubiquinone, is a naturally occurring compound widely distributed in cells, particularly within energy-demanding organs. It plays a central role in mitochondrial ATP synthesis and exhibits potent antioxidant activity, thereby protecting cellular components from oxidative stress [110]. Pharmacologically, CoQ10 supplementation has been applied in conditions associated with mitochondrial dysfunction, oxidative injury, and inflammation, such as cardiovascular diseases, statin-induced myopathy, and neurodegenerative disorders [48]. Experimental evidence further demonstrates its renoprotective potential, as studies have shown that CoQ10 mitigates amiodarone-induced oxidative and inflammatory stress in the kidneys, reducing biochemical alterations and preserving tissue integrity [109].

### 5.3 Grapefruit juice

Grapefruit is a citrus fruit rich in vitamin C, antioxidants, and flavonoids such as naringin, which contribute significantly to its health-promoting properties [111]. Although it is widely recognized for interacting with numerous medications through inhibition of intestinal enzymes such as CYP3A4, emerging evidence highlights its protective effects against drug-induced organ toxicity [112]. Notably, grapefruit juice has been shown to attenuate amiodarone-induced nephrotoxicity by preserving renal architecture, maintaining typical tubular and glomerular structures, and reducing inflammation, degenerative alterations, and proteinaceous casts. Biochemically, it decreases elevated serum creatinine and blood urea nitrogen levels, thereby restoring renal function markers toward normal ranges. Histologically, it replenishes glycogen and protein reserves in renal tissues depleted by amiodarone exposure. Furthermore, grapefruit juice mitigates DNA fragmentation and apoptosis by suppressing oxidative stress mediated by reactive oxygen species. These renoprotective effects are attributed to its phytochemical constituents, including naringin, bergaptol, and geranylcoumarin, which serve as potent antioxidants and modulators of CYP3A4 activity. Collectively, these findings suggest that grapefruit juice exerts a multifaceted protective role and holds preclinical potential in ameliorating amiodarone-induced kidney injury [113].

### 5.4 Grape seed

Grape seed extract (GSE), derived primarily from grape seeds, is a potent source of polyphenols, with approximately 60–70% concentrated within the seeds [114]. It contains phenolic acids, monomeric to trimeric flavanols, and polymeric procyanidins, compounds that collectively underpin its biological activities [49]. GSE has been shown to exert strong antioxidant, antithrombotic, and cardioprotective effects, in addition to modulating inflammatory pathways by reducing proinflammatory cytokines such as IL-6 and TNF-α [115]. Exposure to AMD has been associated with renal injury, including wrinkled basement membranes, damaged mesangial cells, distorted proximal tubules, and elevated biomarkers of injury, oxidative stress, and inflammation. Administration of GSE demonstrated protective effects by improving renal histology and ultrastructure, restoring antioxidant defenses through increased SOD activity and reduced MDA, lowering creatinine levels, and suppressing inflammatory cytokines such as IL-6. Collectively, these findings suggest that GSE protects against AMD-induced nephrotoxicity by attenuating oxidative stress and inflammatory responses [116].

### 5.5 Zizyphus spina christi

Zizyphus spina-christi (ZSC) is a medicinal plant traditionally used to treat disorders of the digestive, hepatic, urinary, respiratory, and immune systems, as well as metabolic and skin conditions [117]. Its leaves are rich in bioactive compounds such as flavonoids, tannins, saponins, and essential oils, which provide antioxidant, anti-inflammatory, hepatoprotective, and immunomodulatory activities [50]. Evidence shows that amiodarone treatment leads to kidney toxicity, reflected in altered biochemical markers (elevated urea and creatinine), increased inflammatory markers (IL-1α, IL-1β, IL-4, IL-6, IL-10, IFN-γ, and IFN-α), and damaged histopathological markers (tubular degeneration, vascular congestion, and hyaline casts). Co-administration of ZSC improves these markers by restoring biochemical balance, reducing inflammatory responses, and preserving renal tissue structure. These findings highlight the potential of ZSC as a natural preclinical candidate for investigation against drug-induced organ toxicity [79].

## 6 Active ingredient against amiodarone-induced thyroid toxicity

### 6.1 Mesenchymal stem cells (MSCs)

Mesenchymal stem cells (MSCs) are multipotent cells with the capacity for self-renewal and differentiation into connective tissue lineages, underscoring their therapeutic relevance in regenerative medicine. They can be derived from diverse tissue sources, and recent evidence supports the presence of adult stem cells within the human thyroid, suggesting a potential role in thyroid tissue repair following injury [118,119]. MSCs demonstrated a significant preclinical potential in mitigating amiodarone-induced thyroid impairment. Experimental findings indicated that MSC administration attenuated histopathological alterations such as follicular degeneration, colloid depletion, and inflammatory infiltration, while partially restoring follicular cell architecture. Functionally, MSCs contributed to the normalization of thyroid hormone levels, characterized by increased T3 and T4, accompanied by a concomitant reduction in TSH. At the molecular level, MSCs exerted antioxidative effects by enhancing SOD and glutathione peroxidase (GPx) activity and reducing MDA levels, thereby alleviating oxidative stress. Furthermore, they modulated apoptotic and inflammatory pathways, as evidenced by the upregulation of the anti-apoptotic marker Bcl-2 and the downregulation of pro-apoptotic and pro-inflammatory markers, including BAX, Caspase-3, IL-6, VEGF, and iNOS. Collectively, these findings underscore the capacity of MSCs to restore thyroid structural integrity and functional homeostasis in the context of amiodarone-induced toxicity [120].

### 6.2 Mesenchymal stem cells (MSCs) with melatonin

Melatonin, a hormone predominantly secreted by the pineal gland, is recognized for its regulatory role in circadian rhythms and energy metabolism, as well as its potent antioxidant, anti-inflammatory, and anti-apoptotic properties [121]. It functions as a free radical scavenger and metal chelator, thereby mitigating oxidative stress, while also stabilizing cellular membranes and attenuating inflammatory responses. Additionally, melatonin modulates apoptotic pathways by downregulating Bax and caspase-3 and upregulating Bcl-2, ultimately promoting cell survival. These protective mechanisms position melatonin as a robust adjunctive candidate for co-treatment with MSCs in regenerative applications [51]. The addition of melatonin to MSC therapy provides superior protection against amiodarone-induced thyroid injury compared to MSC therapy alone. Combined treatment resulted in improved follicular architecture, reduced fibrosis, and restoration of colloid content. It also enhanced antioxidant defenses, reduced oxidative stress and apoptosis, and downregulated inflammatory mediators such as IL-6 and VEGF. Functionally, the therapy normalized thyroid hormones, with increased T3 and T4, and reduced TSH. Melatonin further supported MSC viability and engraftment, underscoring its role as an effective co-therapy in thyroid repair.

## 7 Active ingredient against amiodarone-induced testicular toxicity

### 7.1 Gilaburu

Gilaburu (*Viburnum opulus* L.) (GL), a fruit native to Turkey, contains a rich profile of bioactive constituents, including flavonoids, phenolic compounds, organic acids, and essential vitamins, which collectively underpin its potent antioxidant capacity. These phytochemicals also contribute to its reported antiproliferative, antiallergic, antiviral, and anti-inflammatory activities. Traditionally employed in the management of circulatory, respiratory, reproductive, and digestive disorders, GL has been particularly associated with therapeutic use in rheumatism, hypertension, diabetes, and urinary incontinence, underscoring its broad pharmacological potential [122,123]. GL has been investigated for its potential to alleviate AMD-induced testicular toxicity. AMD administration was associated with marked histopathological alterations, including irregular seminiferous tubules, loss of spermatogenic cells, degeneration, and nuclear pyknosis, alongside reductions in testicular weight, sperm count, and motility, as well as increased TNF-α expression indicative of inflammation. Treatment with GL ameliorated these adverse effects by preserving testicular architecture, improving sperm parameters, and reducing inflammatory responses. The protective activity of GL is attributed to its antioxidant and anti-inflammatory properties, mediated by phenolic constituents such as ellagic acid, catechin, quercetin, and ferulic acid. Collectively, these findings suggest that GL warrants further preclinical evaluation for mitigating AMD-induced reproductive toxicity [124].

## 8 Comparative synthesis and evidence strength

The evidence base was broad but shallow and overwhelmingly preclinical. Pulmonary models predominated, followed by hepatic and renal models; thyroid and reproductive toxicity were represented by few studies. Thirty-four reports were preclinical (animal, in vitro, or mixed-model) and one was an observational human study. No randomized clinical trial evaluated a protective intervention.

Vitamin E had the broadest replication in pulmonary models, although its effects were model- and cell-type-dependent and it did not consistently prevent mitochondrial dysfunction [44,45,106,107,125]. Curcumin showed directionally consistent pulmonary effects in two animal reports [35,87]. Silymarin was supported by two hepatic studies [29,74], and l-carnitine by complementary in vitro and animal evidence [39,95]. Grape-seed preparations were studied in separate hepatic and renal models [59,116]. These interventions therefore have comparatively stronger preclinical support than compounds evaluated in a single study; none has demonstrated clinical efficacy.

Most single-study compounds reduced lipid peroxidation, restored GSH/SOD/catalase, or improved histology. These recurrent downstream outcomes support biological plausibility but do not identify a unique target and are vulnerable to selective outcome reporting. Greater evidentiary weight should be assigned when functional, biochemical, histological, and proximal mechanistic measures—such as tissue drug concentration, phospholipidosis, mitochondrial respiration, or collagen deposition—change coherently.

## 9 Integrated molecular mechanisms

Amiodarone and desethylamiodarone accumulate in lipid-rich compartments. Lysosomal trapping and phospholipid binding promote phospholipidosis, whereas mitochondrial accumulation impairs β-oxidation and oxidative phosphorylation. ATP depletion, membrane depolarization, calcium dysregulation, and excess reactive oxygen species reinforce one another. Oxidative injury activates inflammatory pathways, including TLR4/NF-κB/NLRP3 in selected models, shifts BAX/Bcl-2 and caspase signaling toward apoptosis, and can promote TGF-β/Smad-associated fibroblast activation and collagen deposition. Endoplasmic-reticulum stress is plausible but was not measured consistently enough to be considered an established cross-study mechanism.

**Fig. 2:**
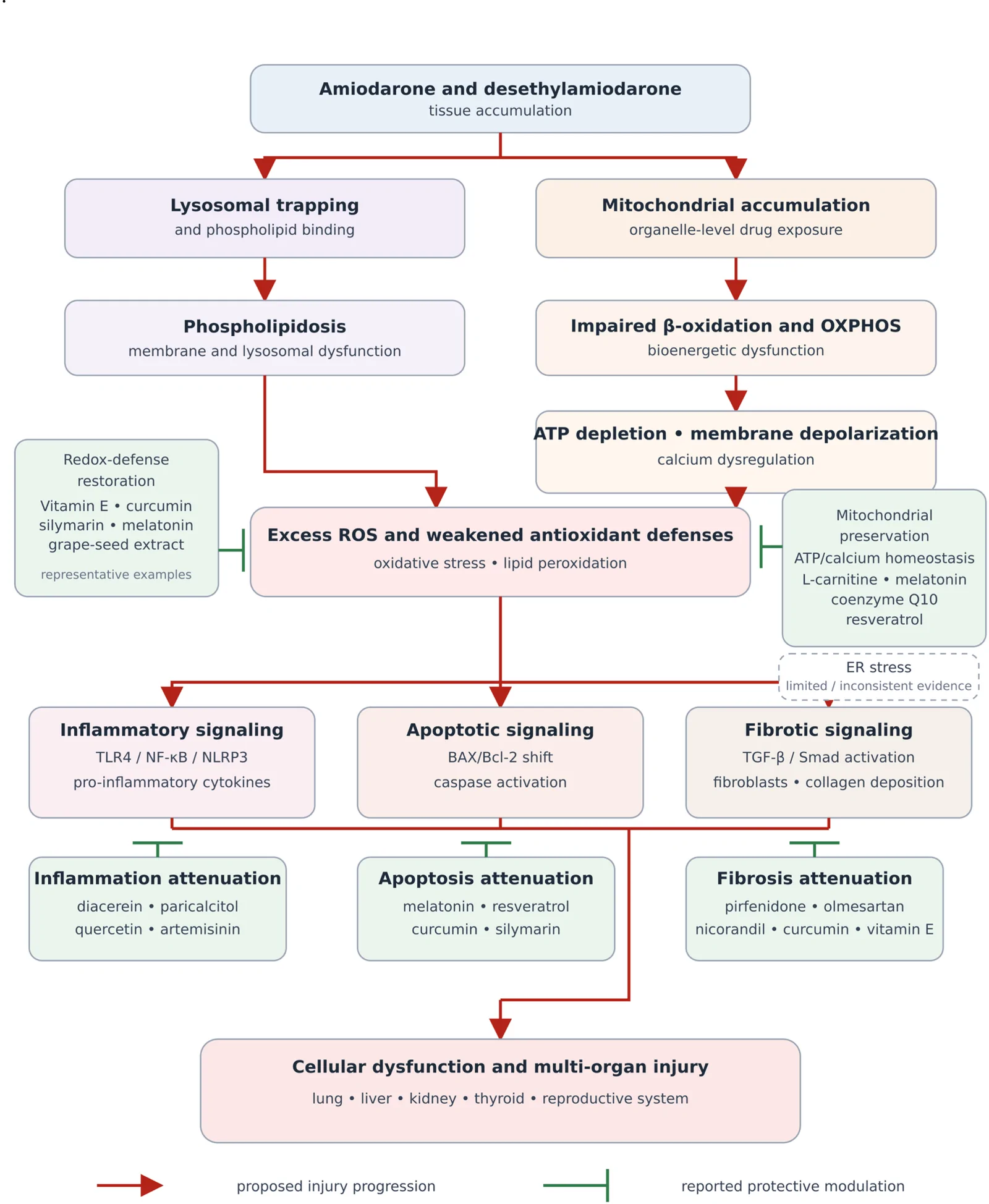
Proposed mechanistic pathways of amiodarone-induced toxicity and reported protective intervention points. Amiodarone and desethylamiodarone accumulate in lysosomes and mitochondria, promoting phospholipidosis, bioenergetic failure, oxidative stress, and inflammatory, apoptotic, and fibrotic signaling. Dashed elements indicate limited or inconsistently measured evidence.

Protective interventions map to four overlapping nodes: restoration of redox defenses; preservation of mitochondrial function and ATP/calcium homeostasis; attenuation of inflammatory signaling; and suppression of apoptosis or fibrosis. Repeated descriptions such as “antioxidant,” “anti-inflammatory,” and “anti-apoptotic” should therefore be interpreted as pathway-level observations rather than proof of distinct molecular targets. The proposed integrated mechanistic framework and reported protective intervention points are summarized in Fig. 2.

## 10 Translational relevance, limitations, and future directions

Clinical translation is premature. Animal amiodarone doses, short prophylactic schedules, and intervention formulations may not reproduce chronic human exposure. Natural products vary in composition and bioavailability and may alter amiodarone pharmacokinetics. Grapefruit juice is a particular concern because CYP3A4 inhibition can change drug exposure; favorable rodent biomarkers do not establish it as a safe adjunct. Repurposed agents such as pirfenidone, nicorandil, paricalcitol, and diacerein are pharmacologically characterized but introduce their own adverse effects and interactions.

The review is limited by English-language restriction, heterogeneous models and outcomes, a small search yield, absence of a prospectively registered protocol, incomplete database-specific search reporting, and insufficient reporting for many risk-of-bias domains. Formal assessment of publication or outcome-reporting bias was not possible because few studies evaluated comparable intervention–outcome combinations and the evidence was synthesized narratively. The predominance of small, positive preclinical studies nevertheless raises concern for publication bias. Heterogeneity also precluded meta-analysis and formal grading of clinical certainty.

Future studies should preregister protocols; justify clinically relevant amiodarone exposure; report randomization, allocation concealment, blinding, and sample-size calculations; measure amiodarone and desethylamiodarone concentrations; include both sexes when appropriate; and use standardized functional and histological endpoints. Independent replication and head-to-head comparisons should precede clinical trials. Early human studies should prioritize pharmacokinetics, safety, and validated organ-specific biomarkers.

## 11 Conclusion

Available evidence does not support routine clinical use of any compound to prevent amiodarone toxicity. Vitamin E has the broadest preclinical replication in pulmonary models, while curcumin, silymarin, and l-carnitine show comparatively coherent but still limited evidence. Pulmonary toxicity has the strongest experimental coverage; renal, thyroid, and reproductive protection remain poorly studied. Independent replication, standardized clinically relevant models, interaction testing, and rigorously designed early-phase human studies are the principal priorities.

## CRediT authorship contribution statement

**Batool Zarei:** Writing – review & editing, Writing – original draft. **Sepideh Elyasi:** Data curation.

## Declaration of generative AI and AI-assisted technologies

The authors declare that no AI technologies were used to generate the text during the preparation of this work except for limited assistance in language editing and error correction.

## Funding

This study was funded by 10.13039/501100004748Mashhad University of Medical Sciences.

**Supplementary Table S1:** Databpdisplayase-specific search strategies

Database/interface	Coverage and final search	Search strategy	Limits
PubMed (National Library of Medicine)	Inception to July 2025	(amiodarone[MeSH Terms] OR amiodarone[Title/Abstract]) AND (toxic*[Title/Abstract] OR injury*[Title/Abstract] OR fibrosis[Title/Abstract] OR phospholipidosis[Title/Abstract]) AND (protect*[Title/Abstract] OR prevent*[Title/Abstract] OR attenuate*[Title/Abstract] OR ameliorate*[Title/Abstract])	English; original research
MEDLINE	Inception to July 2025	amiodarone AND (toxicity OR injury OR fibrosis OR phospholipidosis) AND (protect* OR prevent* OR attenuate* OR ameliorate*)	English; original research
Scopus	Inception to July 2025	TITLE-ABS-KEY(amiodarone AND (toxic* OR injury* OR fibrosis OR phospholipidosis) AND (protect* OR prevent* OR attenuate* OR ameliorate*))	English; article
Web of Science Core Collection	Inception to July 2025	TS=(amiodarone AND (toxic* OR injury* OR fibrosis OR phospholipidosis) AND (protect* OR prevent* OR attenuate* OR ameliorate*))	English; article

## Declaration of competing interest

The authors reported no potential conflict of interest.

## Data Availability

Data will be made available on request.

## Abbreviation

ACE-Is: Angiotensin-Converting Enzyme Inhibitors
AKT: Protein Kinase B
ALP: Alkaline Phosphatase
ALT: Alanine Aminotransferase
AMD: Amiodarone
AMPK: 5′-Amp-Activated Protein Kinase
APT: Amiodarone-Induced Pulmonary Toxicity
ARBs: Angiotensin Receptor Blockers
ARDS: Acute Respiratory Distress Syndrome
ART: Artemisinin
AST: Aspartate Aminotransferase
BUN: Blood Urea Nitrogen
CaMKK2 kinase: Calcium/calmodulin-dependent protein kinase kinase 2
CAT: Catalase
CATe: Catechin
CoQ10: Coenzyme Q10
CPK: Creatine phosphokinase
CYP3A4: Cytochrome P450 3A4
Diacerein: DcN
DNA: Deoxyribonucleic acid
eNOS: Endothelial nitric oxide synthase
Epi: Epicatechin
FA: Ferulic Acid
GA: Gallic Acid
GL: Gilaburu
GPx: Glutathione peroxidase
GSE: Grape seed extract
GSH: Glutathione
γ-GT: Gamma-Glutamyl-Transferase
HIF-1α: Hypoxia-inducible factor-1α
HPE: Human Placenta Extract
IFN-α: Interferon-alpha
IL: Interleukin
IL-1β: Interleukin-1 beta
INOS: Inducible Nitric Oxide Synthase
IPF: Idiopathic Pulmonary Fibrosis
KIM-1: Kidney Injury Molecule-1
LC: l-Carnitine
LDH: Lactate Dehydrogenase
LKB1: Liver kinase B1
LPO: Lipid Peroxidation
MDA: Malondialdehyde
MLT: Melatonin
MRNA: Messenger Ribonucleic Acid
MSCs: Mesenchymal stem cells
mTOR: mechanistic Target of Rapamycin
NF-κB: Nuclear Factor K-Light-Chain-Enhancer Of Activated B Cells
NM: Nutrient mixture
PDGF: Platelet-Derived Growth Factor
Pi3k: Phosphoinositide 3-Kinase
pSmad 3: Phosphorylated mothers against decapentaplegic 3
RAS: Renin-Angiotensin System
ROS: Reactive oxygen species
RV: Resveratrol
SLM: Silymarin
SOD: Superoxide Dismutase
TBARS: Thiobarbituric Acid Reactive Substances
TGF-Β1: Transforming Growth Factor-Β1
TLR4: Toll-like receptor 4
TNF-α: Tumor Necrosis Factor-Alpha
TSH: Thyroid-stimulating hormone
VEGF: Vascular endothelial growth factor
ZSC: Zizyphus Spina-Christi
