# Supplementary material for: The protective effect of chemical and natural compounds against amiodarone induced toxicity: A systematic review
Source: Biochem Biophys Rep. 2026 Sep 6;48:102759. doi: 10.1016/j.bbrep.2026.102759 (PMC13578413; doi:10.1016/j.bbrep.2026.102759)
Supplement: Supplementary file 1 [file mmc1.docx]

**PRISMA 2020 Checklist**

**The Protective Effect of Chemical and Natural Compounds Against Amiodarone Induced Toxicity: A Systematic Review**

| Section and topic | Item | Reporting requirement (abridged) | Location in revised manuscript |
| --- | --- | --- | --- |
| TITLE — Title | **1** | Identify the report as a systematic review. | p. 1, lines 1–2. |
| ABSTRACT — Abstract | **2** | Provide the information required by the PRISMA 2020 for Abstracts checklist. | Structured abstract: p. 2, lines 33–59. |
| INTRODUCTION — Rationale | **3** | Explain the rationale in the context of existing knowledge. | pp. 4–5, lines 83–125. |
| INTRODUCTION — Objectives | **4** | State the review objective(s) or question(s) explicitly. | p. 5, lines 126–128. |
| METHODS — Eligibility criteria | **5** | Specify inclusion and exclusion criteria and how studies were grouped for synthesis. | pp. 6–7, lines 155–167; synthesis grouping on pp. 7–8, lines 183–193. |
| METHODS — Information sources | **6** | List databases and other sources and report when each was last searched. | p. 6, lines 145–149; Supplementary Table S1, p. 40 (caption at line 1262). |
| METHODS — Search strategy | **7** | Present the complete search strategies, including filters and limits. | Main concepts: p. 6, lines 145–154; full database-specific strategies: Supplementary Table S1, p. 40 (caption at line 1262). |
| METHODS — Selection process | **8** | Describe how reviewers decided whether records met inclusion criteria. | p. 7, lines 168–175. |
| METHODS — Data collection process | **9** | Describe how data were collected, including reviewer independence and resolution of disagreements. | p. 7, lines 176–184. |
| METHODS — Data items: outcomes | **10a** | Define the outcomes sought and how compatible results were handled. | pp. 6–7, lines 159–160 and 177–182; handling of heterogeneous outcomes: pp. 7–8, lines 184–193. |
| METHODS — Data items: other variables | **10b** | Define other variables collected and assumptions about missing or unclear information. | p. 7, lines 177–182. No additional assumptions or imputations are reported. |
| METHODS — Risk of bias | **11** | Describe the tools, domains, reviewers, and process used to assess study-level risk of bias. | p. 8, lines 202–215. |
| METHODS — Effect measures | **12** | State the effect measure used for each outcome. | results were summarized by direction of change: pp. 7–8, lines 189–193; Table 1, pp. 40–44 (caption at line 1265). |
| METHODS — Synthesis: eligibility | **13a** | Describe how studies were judged eligible for each synthesis. | Eligibility: pp. 6–7, lines 155–167; synthesis grouping: pp. 7–8, lines 183–193. |
| METHODS — Synthesis: data preparation | **13b** | Describe preparation of data for presentation or synthesis. | Direction-of-change summaries were used; effect magnitudes were not compared: p. 8, lines 189–193. No imputation or conversion is reported. |
| METHODS — Synthesis: display | **13c** | Describe methods used to tabulate or visually display study results. | Narrative organization: pp. 7–8, lines 183–193; Table 1, pp. 40–44 (caption at line 1265); Table 2, p. 44 (caption at line 1268). |
| METHODS — Synthesis: methods | **13d** | Describe the synthesis methods and justify the approach. | Narrative synthesis rationale and method: pp. 7–8, lines 183–193; comparative synthesis: p. 28, lines 777–795. No meta-analysis was performed. |
| METHODS — Synthesis: heterogeneity | **13e** | Describe methods used to explore possible causes of heterogeneity. | Heterogeneity was addressed qualitatively by organ, model, intervention, mechanism, replication, and outcome direction: pp. 7–8, lines 183–193; p. 28, lines 777–795. |
| METHODS — Synthesis: sensitivity | **13f** | Describe sensitivity analyses used to test robustness. | Not performed because no quantitative meta-analysis was undertaken (pp. 7–8, lines 183–193). |
| METHODS — Reporting bias assessment | **14** | Describe methods used to assess bias from missing results. | pp. 8–9, lines 216–221. |
| METHODS — Certainty assessment | **15** | Describe any method used to assess certainty or confidence in the evidence. | p. 28, lines 777–795; Table 2, p. 44 (lines 1268–1270). |
| RESULTS — Study selection | **16a** | Report selection results, preferably with a flow diagram. | Selection counts: p. 8, lines 194–201; Figure 1 caption: p. 9, line 222. |
| RESULTS — Excluded studies | **16b** | Cite studies that appeared eligible but were excluded and explain why. | p. 8, lines 196–201; therefore, no excluded full-text citation list was required. |
| RESULTS — Study characteristics | **17** | Cite each included study and present its characteristics. | Organ-specific Results: pp. 9–28, lines 228–776; Table 1, pp. 40–44 (caption at line 1265). |
| RESULTS — Risk of bias in studies | **18** | Present risk-of-bias assessments for included studies. | Methods and narrative findings: p. 8, lines 202–215; the supplementary risk-of-bias table is referenced at line 210. |
| RESULTS — Individual-study results | **19** | For each study, present outcome results and available effect estimates and precision. | Study-reported results: pp. 9–28, lines 228–776; Table 1, pp. 40–44 (caption at line 1265). Common effect estimates and precision were not calculated because measures were heterogeneous. |
| RESULTS — Syntheses: contributors | **20a** | Summarize characteristics and risk of bias among studies contributing to each synthesis. | Comparative synthesis: p. 28, lines 777–795; Table 2, p. 44 (lines 1268–1270). |
| RESULTS — Syntheses: statistical results | **20b** | Present results of statistical syntheses and their precision and heterogeneity. | Narrative comparative results: p. 28, lines 777–795. |
| RESULTS — Syntheses: heterogeneity | **20c** | Present investigations of possible causes of heterogeneity. | Qualitative comparison by model, organ, replication, and mechanism: p. 28, lines 777–795; limitations: p. 29, lines 818–824. |
| RESULTS — Syntheses: sensitivity | **20d** | Present sensitivity-analysis results. | Not applicable; no meta-analysis or sensitivity analysis was performed. |
| RESULTS — Reporting biases | **21** | Present assessments of bias due to missing results. | Qualitative assessment: pp. 8–9, lines 216–221; publication-bias concern and limitations: p. 29, lines 821–824. |
| RESULTS — Certainty of evidence | **22** | Present certainty or confidence assessments for each outcome. | Descriptive evidence strength: p. 28, lines 777–795; Table 2, p. 44 (lines 1268–1270). |
| DISCUSSION — Interpretation | **23a** | Interpret results in the context of other evidence. | Comparative interpretation and integrated mechanisms: pp. 28–29, lines 777–809. |
| DISCUSSION — Evidence limitations | **23b** | Discuss limitations of the evidence included in the review. | p. 29, lines 811–824. |
| DISCUSSION — Review-process limitations | **23c** | Discuss limitations of the review processes used. | p. 29, lines 818–824. |
| DISCUSSION — Implications | **23d** | Discuss implications for practice, policy, and future research. | pp. 29–30, lines 825–837. |
| OTHER — Registration | **24a** | Provide registration details or state that the review was not registered. | this limitation is acknowledged on p. 29, lines 818–820. |
| OTHER — Protocol | **24b** | State where the protocol can be accessed or that none was prepared. | No prospectively registered or publicly accessible protocol is reported; p. 29, lines 818–820. |
| OTHER — Amendments | **24c** | Describe and explain amendments to registration or protocol. | Not applicable/not reported because no prospective registration or public protocol was available (p. 29, lines 818–820). |
| OTHER — Support | **25** | Describe financial and nonfinancial support and the role of funders. | Acknowledgments: p. 30, lines 848–850; Funding: p. 31, lines 851–852. The funder’s role is not stated. |
| OTHER — Competing interests | **26** | Declare competing interests. | p. 30, lines 843–844. |
| OTHER — Availability | **27** | Report public availability of forms, extracted data, analysis data/code, and other review materials. | Data Availability Statement: p. 30, lines 838–841. Extraction and risk-of-bias tables are identified for supplementary provision were permitted |
